# Efficacy of a Novel Prophylactic Scheme of Fosfomycin Trometamol in Patients Undergoing Endoscopic Surgery for Benign Prostatic Hyperplasia: Findings from a Prospective Monocentric Single-Arm Study

**DOI:** 10.3390/antibiotics13050424

**Published:** 2024-05-06

**Authors:** Pasquale Maria Berrino, Milo Gatti, Valeria Rotaru, Lorenzo Bianchi, Fabio Tumietto, Elena Sora, Riccardo Schiavina, Eugenio Brunocilla, Pierluigi Viale, Federico Pea

**Affiliations:** 1 Division of Urology, IRCCS Azienda Ospedaliero-Universitaria of Bologna, 40138 Bologna, Italy; pasquale.berrino@studio.unibo.it (P.M.B.); valeria.rotaru2@studio.unibo.it (V.R.); lorenzo.bianchi13@unibo.it (L.B.); riccardo.schiavina3@unibo.it (R.S.); eugenio.brunocilla@unibo.it (E.B.); 2 Department of Medical and Surgical Sciences, Alma Mater Studiorum University of Bologna, 40138 Bologna, Italy; pierluigi.viale@unibo.it (P.V.); federico.pea@unibo.it (F.P.); 3 Clinical Pharmacology Unit, Department for integrated Infectious Risk Management, IRCCS Azienda Ospedaliero-Universitaria of Bologna, 40138 Bologna, Italy; 4 Antimicrobical Stewardship Unit, Department for integrated Infectious Risk Management, Azienda USL of Bologna, 40138 Bologna, Italy; fabio.tumietto@ausl.bologna.it (F.T.); e.sora@ausl.bologna.it (E.S.); 5 Infectious Disease Unit, Department for Integrated Infectious Risk Management, IRCCS Azienda Ospedaliero-Universitaria of Bologna, 40138 Bologna, Italy

**Keywords:** fosfomycin, antibiotic prophylaxis, urological procedures, benign prostatic hyperplasia, clinical efficacy

## Abstract

This study aimed to assess the efficacy of a novel prophylactic scheme of fosfomycin trometamol in patients undergoing elective HoLEP (holmium laser enucleation of the prostate) or TURP (transurethral resection of the prostate) procedures for treating benign prostatic hyperplasia. Patients affected by benign prostatic hyperplasia and undergoing elective HoLEP or TURP procedures during the period February 2022–June 2023 were prospectively enrolled. Two 3 g oral fosfomycin trometamol doses 12 h apart were administered at 8.00 p.m. on day −1 (i.e., the day before HoLEP or TURP procedure) and at 8.00 a.m. on day 0 (i.e., the day of the surgical procedure). The following outcomes were assessed: prevalence of fever occurring in the first 48 h after surgical procedure; prevalence of urological complications occurring after the surgical procedure; prevalence of proven urinary tract infections (UTIs) and/or bloodstream infections (BSIs) at 14 days post-procedure; and prevalence of emergency department admission for UTI-related sepsis at 14 days post-procedure. Univariate analysis comparing patients with and without proven UTI, BSI, or emergency department admission at 14 days post-procedure was carried out. Overall, 96 patients (median age 70 years) undergoing HoLEP (82.3%) or TURP (17.7%) were prospectively included. Median (IQR) time of surgical procedure after the morning fosfomycin dose was 226.5 min (range 88.5–393.75 min). Fever in the post-surgical 48 h occurred in 3/96 patients (3.1%). Prevalence of proven UTI at 14 days was as low as 1.0% (1/96), whereas no patient had proven BSI or UTI-related sepsis requiring emergency department admission at 14 days. Our findings support the contention that a prophylactic scheme based on two doses of fosfomycin trometamol 12 h apart before surgical intervention may represent a valuable strategy for preventing infectious complications in urologic patients undergoing HoLEP or TURP. Larger definitive confirmatory studies are warranted.

## 1. Introduction

Benign prostatic hyperplasia (BPH) represents a common condition affecting males aged over 50 years, with an estimated prevalence of almost 80% in those aged >70 years [1]. Benign prostatic hyperplasia is caused by unregulated tissue proliferation within the prostate, resulting in physical obstruction of the prostatic tract of the urethra and consequent obstruction in the anatomic bladder outlet [1].

Transurethral resection of the prostate (TURP) has historically represented the gold standard surgical procedure for managing benign prostatic hyperplasia [2]. However, holmium laser enucleation of the prostate (HoLEP) has recently emerged as a novel endoscopic technique for minimally invasive surgery for BPH. Indeed, HoLEP may have several advantages compared to TURP, such as better management of voluminous prostates and improved hemostasis. Moreover, due to fewer post-operative complications, shorter catheter time and hospital stays, HoLEP has replaced open prostatectomy in most high-volume centers treating BPH [3].

Both TURP and HoLEP are classified as clean-contaminated interventions carrying an infectious risk of 4–10%, so that antibiotic prophylaxis is needed [4]. Both the American Urological Association and the European Association of Urology recommend for this purpose an oral fluoroquinolone or cotrimoxazole with the intent of providing effective coverage against the *Enterobacterales* (particularly *Escherichia coli*) [5,6]. Unfortunately, the worryingly increasing prevalence of resistance rates among the *Enterobacterales* to both of these traditional prophylactic agents imposes the need for alternative choices [7,8].

Fosfomycin trometamol is an oral antibiotic showing valuable coverage against several types of multi-drug resistant (MDR) pathogens, including extended-spectrum beta-lactamase (ESBL)-producing *Enterobacterales* [9]. From a pharmacokinetic standpoint, fosfomycin trometamol has good oral bioavailability and a high volume of distribution with negligible plasma protein binding, high penetration rates into deep tissues, including bone, central nervous system, lung, and prostate, and it is eliminated from the body as an unchanged moiety via the renal route [10,11,12,13]. Generally, fosfomycin trometamol is well-tolerated, and adverse events (mainly self-limiting) have been reported in approximately 1–10% of patients [10,11,12]. Some studies have shown that fosfomycin trometamol may be more effective compared to fluoroquinolones as antibiotic prophylaxis in urological patients, resulting in lower risk of both symptomatic urinary tract infections (UTI) and urosepsis in the first two weeks after a surgical procedure [14,15]. Additionally, fosfomycin may also represent a valuable fluoroquinolone-sparing strategy, considering the recently emerged safety and ecological concerns associated with the use of these agents [16,17]. Other studies have previously evaluated the efficacy of fosfomycin in the urological setting and adopted a prophylactic/pre-emptive strategy, administering one 3 g dose 3 h before the intervention and another 24 h after. However, it has been shown that attaining fosfomycin therapeutic concentrations in prostatic tissue may take some hours after oral administration [18]. Consequently, it could be hypothesized that this scheme could expose urological patents undergoing intervention in the first hours after the first dose to the risk of suboptimal prostatic target attainment. In this scenario, adopting a novel fully prophylactic regimen of fosfomycin based on two 12 h apart pre-intervention doses could minimize this risk. Additionally, this novel scheme could have the theoretical advantage of abating any eventual bacterial load associated with an asymptomatic bacteriuria that could occur in the 12 h before the intervention.

According to these assumptions, the aim of this study was to assess the efficacy of this novel scheme of antibiotic prophylaxis with oral fosfomycin trometamol in patients undergoing elective HoLEP or TURP procedures for BPH.

## 2. Results

**Figure 1:**
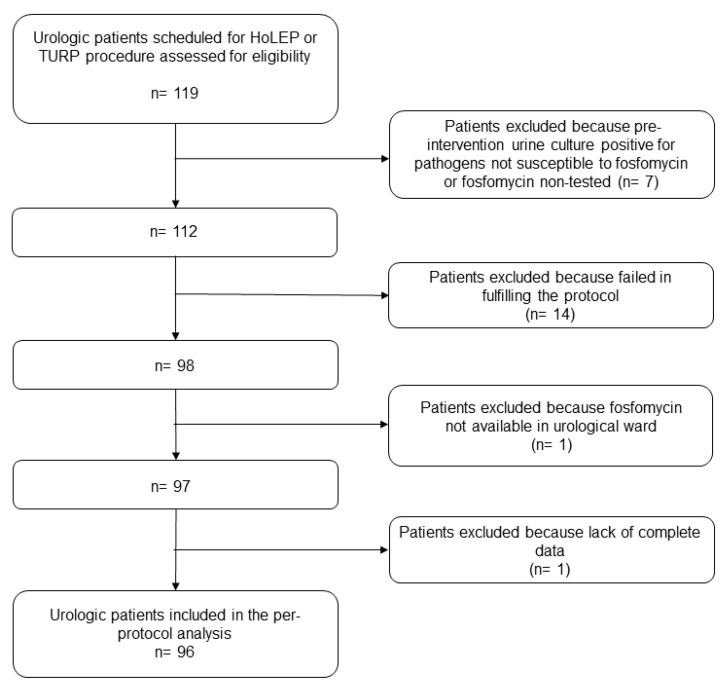
Flowchart of patient inclusion and exclusion criteria. HoLEP: holmium laser enucleation of the prostate; TURP: transurethral resection of the prostate.

From February 2022 to June 2023, a total of 119 urological patients undergoing HoLEP or TURP for BPH were screened for eligibility. Among these, 96 were included in the per-protocol analysis (excluded cases: 14 because of protocol non-adherence, 7 because of pre-intervention positivity of urine cultures, and 1 each because of fosfomycin unavailability or incomplete data) (Figure 1).

Demographics and clinical features of the 96 included patients are reported in Table 1.

The median (IQR) age was 70 years (66–76 years). The median (IQR) CCI score was 4 points (2–4 points), and immunodepression was present in 13.5% of cases. The most prevalent underlying comorbidities were arterial hypertension (61.5%), dyslipidemia (33.3%), and diabetes mellitus (19.8%).

The median (IQR) baseline CLCr was 79 mL/min/1.73 m^2^ (69.75–86 mL/min/1.73 m^2^). One patient had chronic renal failure requiring IHD, whereas no patients experienced ARC.

At pre-intervention transrectal prostatic ultrasound, the overall median (IQR) prostate volume was 76 cc (50–99.5 cc), with a median (IQR) prostate adenoma volume of 46 cc (34–55 cc). The median (IQR) baseline serum PSA level was 3.05 ng/mL (1.35–5.67 ng/mL).

HoLEP and TURP were carried out in 79 (82.3%) and 17 (17.7%) cases, respectively. The median (IQR) elapsed time from second fosfomycin dose administration to intervention was 226.5 min (88.5–393.75 min). Twenty-one patients (21.9%) underwent HoLEP or TURP within 60 min after receiving the second fosfomycin dose.

Three patients (3.1%) had fever in the first 48 h post HoLEP, all with normal white blood cells count and no urinary signs or symptoms. None of these patients received antibiotic therapy during their hospital stay or at discharge. Ten patients had post-surgical urological complications (nine acute urinary retention with bladder catheter placement, and one macrohematuria). Only one patient (1.0%) had a proven UTI occurring 10 days after the surgical procedure (due to a full-susceptible *Klebsiella pneumoniae*), with no requirement for hospital admission. No patient had proven BSI or UTI-related sepsis requiring ED admission at 14 days. Univariate analysis comparing patients having or not having post-surgical documented infections was unfeasible due to the poor occurrence of events.

## 3. Materials and Methods

### 3.1. Study Design

This single center prospective study was carried out between 1 February 2022 and 30 June 2023 in the urological ward of the “Istituto di Ricovero e Cura a Carattere Scentifico” (IRCCS) Azienda Ospedaliero-Universitaria of Bologna, Italy. The study was conducted according to the guidelines of the Declaration of Helsinki and approved by the local ethical committee (No. 882/2021/Oss/AOUBo on 31 January 2022). Signed informed consent was collected from each included patient.

**Figure 2:**
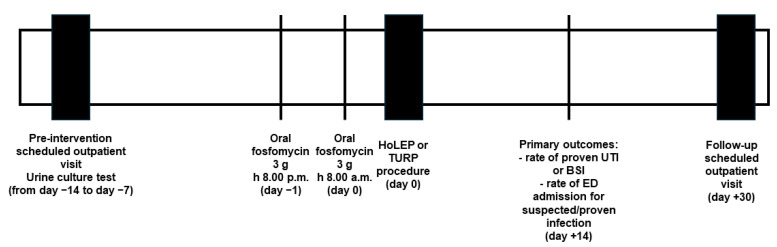
Flowchart of the study design. BSI: bloodstream infection; ED: emergency department; HoLEP: holmium laser enucleation of the prostate; TURP: transurethral resection of the prostate; UTI: urinary tract infection.

A summary of the study design is reported in Figure 2. All potentially eligible patients performed a pre-intervention scheduled urological outpatient visit (i.e., in the two weeks before HoLEP or TURP procedure), coupled with a urine culture test. Included patients undergoing elective HoLEP or TURP received antimicrobial prophylaxis with two doses of 3 g oral fosfomycin trometamol 12 h apart, namely at 8.00 p.m. on the day before the surgical procedure, and at 8.00 a.m. on the day of the surgical procedure. HoLEP or TURP was executed within the end of the same morning. A positive pre-procedural urine culture test or placement of bladder catheter in the pre-procedural period represented the exclusion criteria.

Oral fosfomycin was self-administered or administered by the attending hospital personnel depending on whether the participant was an outpatient or an inpatient, respectively.

Clinical outcome was assessed as follows: at 48 h, rate of post-surgical fever occurrence and urological complications; at 14 days, rate of proven urinary tract infections [UTIs] and/or of bloodstream infections [BSIs] and/or of UTI-related sepsis requiring emergency department admission; at 30 days, clinical status as outpatient.

### 3.2. Surgical Procedures

Patients with complicated BPH, or who were non-respondent to pharmacological treatment, were scheduled for surgical treatment. The choice between TURP and HoLEP was determined by the treating urologist according to preoperative prostatic volume measured by transrectal prostatic ultrasound performed within 6 months before surgery. Patients with prostate volume ≤ 70 cc were scheduled for TURP, whereas those with prostate volume > 70 cc were scheduled for HoLEP [19].

Preoperative assessment of patients eligible for surgery consisted of: prostate-specific antigen (PSA) dosage, digital rectal examination (DRE), uroflowmetry, the use of international questionnaires, including International Prostatic Symptoms Score (IPSS) and Quality of Life (QoL), an abdominal ultrasound to evaluate post-voiding residual volume and hydronephrosis, and a transrectal ultrasound to evaluate prostatic volume.

In case of suspected prostate cancer (according to elevated PSA, PSA density, DRE, and familial history), patients underwent multiparametric magnetic resonance imaging (MRI) and transperineal prostatic biopsy (target and/or systematic biopsy) for detecting suspected prostatic lesions [20]. Patients with clinically significant prostatic cancer (i.e., according to the grading consensus conference of the International Society of Urological Pathology [ISUP] ≥ 2) were excluded from the study.

TURP and HoLEP were performed under general or spinal anesthesia. TURP was carried out using a 26 Fr continuous-flow Storz bipolar resectoscope (ESC Medicams, Delhi, India), as previously described [21]. HoLEP was performed using a Lumenis Versa Pulse^®^ Holmium laser (Boston Scientific, St. Paul, MN, USA) at 2.0 J and 50 pulses per second with a maximum average power of 100 W and a 26 Fr continuous-flow Storz laser resectoscope, as previously described [22]. Laser energy was delivered with a 550 μm fiber. The enucleation of prostatic adenoma was performed according to Gilling’s technique [23]. The enucleated prostatic lobes were removed using a Lumenis Versa Cut™ Morcellator System (Boston Scientific, St. Paul, MN, USA).

A 22 Fr three-way catheter was positioned at the end of both procedures with continuous irrigation.

### 3.3. Data Collection

Demographic data (age, sex, weight, height, and body mass index [BMI]), clinical/laboratory data (Charlson Comorbidity Index [CCI], comorbidities, immune status, baseline serum creatinine and creatinine clearance [CLCr], need for intermittent hemodialysis [IHD], occurrence of augmented renal clearance [ARC], overall prostate volume and volume of prostate adenoma at transrectal prostatic ultrasound, pre-intervention serum PSA levels, Uroflowmetry, and IPSS and QoL score), and surgical data (type of scheduled intervention, and time elapsed between administering the second fosfomycin dose and performing the surgical procedure) were collected.

Immune status was defined as depressed whenever one or more of the following conditions were present: need for long-term use of corticosteroids and/or of biologic and/or antineoplastic agents, occurrence of solid or hematologic malignancies, previous solid organ (SOT) or hematopoietic stem cell transplantation (HSCT), or underlying HIV disease or autoimmune disease [24].

ARC was defined as a measured (based on 24 h urine collection) or an estimated (according to the CDK-EPI formula) creatinine clearance above 130 mL/min and 120 mL/min in males and females, respectively [25].

### 3.4. Definition of Outcome Variables

Fever was defined as a body temperature ≥ 37.5 °C on at least one occasion. Post-surgical urological complications were defined as the occurrence of acute urinary retention requiring bladder catheter placement and/or of macrohematuria. Proven UTI was defined as the presence of local and systemic signs and/or symptoms coupled with the isolation from urine of a pathogen with a bacterial load ≥10^5^ CFU/mL at the culture test [26]. Proven BSI was defined as the isolation of a pathogen from at least one blood culture [26]. UTI-related sepsis requiring ED admission was defined based on the following criteria: presence of probable or possible symptoms of UTI (i.e., dysuria, urinary frequency, urgency, hesitancy, urinary retention, difficulty passing urine, hematuria, and malodorous urine) coupled with positive urinary culture at >10^5^ CFU/mL or at >10^3^ CFU/mL plus urinary white cell count >80 cells/µL, or alternatively, isolation of the same pathogen from both the blood and the urine culture [27].

### 3.5. Statistical Analysis

For descriptive analysis, categorical variables were described as absolute values and percentages, whereas continuous variables were reported as means ± standard deviations (SD) or medians and interquartile ranges (IQR), according to the distribution.

Comparison of patients having or not having proven UTI, BSI, or emergency department admission at 14 days post-procedure was carried out by means of chi-square tests or the Fisher exact test as appropriate for categorical variables, and using the Student’s *t* test or Mann–Whitney U test for continuous variables. *p* values < 0.05 were defined as statistically significant. Statistical analyses were performed by means of MedCalc for Windows (MedCalc statistical software Ltd., version 19.6.1, Ostend, Belgium).

## 4. Discussion

To the best of our knowledge, this prospective study was the first to explore the role of a novel prophylactic scheme based on two oral fosfomycin trometamol doses 12 h apart among patients with BPH undergoing HoLEP or TURP. Overall, the findings showed that this prophylactic scheme was an effective strategy, since the prevalence of post-procedural infectious complications was very low.

Our findings are consistent with those of other studies carried out among urologic patients undergoing TURP receiving different dosing schemes of fosfomycin trometamol, either as prophylaxis or as mixed prophylaxis/preemptive [14,28,29,30,31,32,33,34]. In a prospective, randomized, placebo controlled, double-blind study carried out among patients undergoing TURP, two doses of fosfomycin trometamol or of placebo 24 h apart were administered, one in the evening before and the other in the evening after the intervention [28]. The early post-operative prevalence of UTIs was significantly lower among patients receiving fosfomycin (n = 31) than among those receiving the placebo (n = 30) (0.0% vs. 20.0%; *p* = 0.015) [28]. In another prospective, multicentric study enrolling 712 patients undergoing surgical transurethral procedures or urological procedures, two 3 g doses of fosfomycin trometamol were administered, one 3 h before and the other 24 h after the intervention [30]. The prevalences of UTIs 2 and 7 days after the procedure were 3.2% and 3.6%, respectively [30]. In a prospective, controlled, multicentric study, 675 patients undergoing TURP were randomized to receive antibiotic prophylaxis with either amoxycillin (N = 207), cotrimoxazole (N = 212), or fosfomycin trometamol (N = 256) [29]. In this study, two 3 g fosfomycin trometamol doses were again administered, one 3 h before and the other 24 h after the intervention [29]. The risk of postoperative bacteriuria and that of symptomatic UTIs were found to be significantly lower among those receiving fosfomycin (14.8% and 1.9%, respectively) compared to those receiving amoxicillin (24.6% and 8.6%, respectively; *p* < 0.01) or cotrimoxazole (25.0% and 8.4%, respectively; *p* < 0.01) [29]. In a retrospective, multicentric, Italian study including 1109 patients undergoing transrectal ultrasound-guided prostate biopsy, antibiotic prophylaxis/preemptive was based on fosfomycin trometamol (two 3 g doses, one 3 h before and the other 24 h after the intervention) in 632 patients, and on oral ciprofloxacin in the other 477 (500 mg twice daily for five days, starting 24 h before the intervention) [32]. Overall, patients receiving fosfomycin had significantly lower prevalence of both symptomatic UTIs (1.6% vs. 12.9%; *p* < 0.001) and urosepsis (0.3% vs. 1.8%; *p* < 0.001) [32].

Overall, these studies suggest that defining proper timing for fosfomycin administration in prostatic interventions may still represent an arguable topic [35]. In regard to the fosfomycin dosing regimen most frequently adopted in these studies, namely that based on one dose 3 h before and the other 24 h after the intervention [29,30,31,32], some issues may arise. First, strictly speaking, this scheme must be considered as a mixed prophylactic/preemptive strategy. Second, according to a recent population pharmacokinetic model conducted among 26 subjects undergoing TURP, the optimal timeframe for administering fosfomycin prophylaxis should range between 1 and 4 h before the intervention [18,35,36]. This is due to the fact that the attainment of therapeutic concentrations with fosfomycin in the prostate gland may be delayed and blunted compared to those in plasma. Interestingly, the authors also found that 12 h after fosfomycin administration, the concentrations in the transitional prostate zone were still above the MIC_50_ value against *Escherichia coli* in approximately 80% of cases [18]. These findings may support the contention that administering two prophylactic fosfomycin doses 12 h apart before the intervention, as we did, may minimize the likelihood of attaining only subtherapeutic prostatic concentrations among patients undergoing intervention early after the morning prophylactic dose. This novel prophylactic scheme could allow more flexibility in the need for timing variations in planning surgical interventions due to logistic issues. Interestingly, approximately one-quarter of our cohort patients underwent HoLEP or TURP within 60 min after receiving the second fosfomycin dose, and overall had no infections. In this regard, it cannot be ruled out that the prophylactic/preemptive fosfomycin-based regimen tested in previous studies [29,30,31,32] could fail in attaining therapeutic prostatic concentrations in this scenario, thus potentially exposing patients to infection risk. Additionally, our novel prophylactic scheme may have the theoretical advantage of abating any eventual bacterial load associated with an asymptomatic bacteriuria that could occur immediately before the intervention.

Finally, it is noteworthy that adopting a prophylactic strategy based on oral fosfomycin in urologic patients may be worthwhile in terms of antimicrobial stewardship, particularly considering, on the one hand, the ever-growing increase of multi-drug resistance among *Enterobacterales* causing UTIs and, on the other hand, the safety and efficacy concerns regarding fluoroquinolones in this scenario [15].

The limitations of our study should be acknowledged. The monocentric study design and the lack of a control and/or a comparator group must be recognized as major limits preventing us from drawing firm conclusions about the clinical efficacy of this novel fosfomycin trometamol prophylactic regimen. In this regard, our findings are encouraging when looking at a review summarizing the findings of historical studies based on either other types of fosfomycin-based or non-fosfomycin based prophylactic/preemptive regimens [14]. Compared to the former, occurrence rates of fever in the 48 h post intervention were similar (3.1% vs. 3.0%), and the prevalence of symptomatic UTI within 7–14 days was even lower (1.0% vs. 3.3%) [14]. Compared to the latter, lower prevalence rates were reported both regarding fever in the 48 h post intervention (3.1% vs. 4.9–6.0%) and symptomatic UTIs within 7–14 days (1.0% vs. 8.1–8.3%) [14]. Notably, the prospective study design of the novel prophylactic dosing scheme is a point of strength.

## 5. Conclusions

Our findings support the contention that a prophylactic scheme based on two doses of fosfomycin trometamol 12 h apart before surgical intervention may represent a valuable strategy for preventing infectious complications in urologic patients undergoing HoLEP or TURP. Larger definitive confirmatory studies are warranted.

## Figures and Tables

**Table 1:** Demographics and clinical characteristics of the included patients receiving fosfomycin prophylaxis before scheduled HoLEP or TURP.

Demographics and Clinical Variables	Patients (N = 96)
Patient demographics	
Age (years) [median (IQR)]	70 (66–76)
Body weight (Kg) [median (IQR)]	79 (74.0–87.3)
Body mass index (Kg/m^2^) [median (IQR)]	26.7 (24.9–29.3)
Underlying conditions	
Charlson Comorbidity Index [median (IQR)]	4 (2–4)
Arterial hypertension [n (%)]	59 (61.5)
Dyslipidemia [n (%)]	32 (33.3)
Diabetes mellitus [n (%)]	19 (19.8)
Chronic obstructive pulmonary disease [n (%)]	14 (14.6)
Atrial fibrillation [n (%)]	11 (11.5)
Acute myocardial infarction [n (%)]	10 (9.3)
Gastroesophageal reflux [n (%)]	9 (9.4)
Ischemic/Hemorrhagic stroke [n (%)]	6 (6.3)
Solid cancer [n (%)] ^a^	4 (4.2)
Peptic ulcer [n (%)]	4 (4.2)
Chronic renal disease [n (%)]	2 (2.1)
Hematological malignancies [n (%)]	1 (1.0)
Immunosuppression [n (%)]	13 (13.5)
Status of renal function	
Baseline CL_CR_ (mL/min/1.73 m^2^) [median (IQR)]	79 (69.75–86)
IHD [n (%)]	1 (1.0)
Augmented renal clearance [n (%)]	0 (0.0)
Urological conditions before intervention	
Overall prostate volume (cc) [median (IQR)]	76.0 (50.0–99.5)
Prostate adenoma volume (cc) [median (IQR)]	46 (34–55)
Prostate-specific antigen (ng/mL) [median (IQR)]	3.05 (1.35–5.67)
Surgical procedure	
HoLEP [n (%)]	79 (82.3)
TURP [n (%)]	17 (17.7)
Elapsed time from 2nd fosfomycin dose to intervention (minute) [median (IQR)]	226.5 (88.5–393.75)
Outcome	
Fever in the 48-h post procedure [n (%)]	3 (3.1)
Urological complications [n (%)] ^b^	10 (10.4)
14-day proven UTI [n (%)]	1 (1.0)
14-day proven BSI [n (%)]	0 (0.0)
14-day UTI-related sepsis requiring ED admission [n (%)]	0 (0.0)

BSI: bloodstream infection; CL_CR_: creatinine clearance; ED: emergency department; HoLEP: holmium laser enucleation of the prostate; IHD: intermittent hemodialysis; IQR: interquartile range; TURP: transurethral resection of the prostate; UTI: urinary tract infection. ^a^ none of included patients had prostatic cancer. ^b^ acute urinary retention with bladder catheter placement (n = 9), macrohematuria (n = 1).

## Data Availability

The data presented in this study are available on request from the corresponding author. The data are not publicly available due to privacy concerns.
